# Acute Structural, Functional, and Biochemical Responses to a High- and Low-Volume Foam Rolling Exercise on the Hamstring Muscles: A Randomized Controlled Trial

**DOI:** 10.3390/sports14080354

**Published:** 2026-08-17

**Authors:** Jakob Hofer, David G. Behm, Masatoshi Nakamura, Andreas Konrad

**Affiliations:** 1 Institute of Human Movement Science, Sport and Health, University of Graz, 8010 Graz, Austria; jakobhofer92@gmail.com; 2 School of Human Kinetics and Recreation, Memorial University of Newfoundland, St. John’s, NL A1C 5S7, Canada; dbehm@mun.ca; 3 Faculty of Rehabilitation Sciences, Nishi Kyushu University, 4490-9 Ozaki, Kanzaki-shi 842-8585, Saga, Japan; nakamuramas@nisikyu-u.ac.jp

**Keywords:** foam rolling, creatine kinase, MVIC, muscle stiffness, ROM, high-volume, dose response, hamstring, regeneration, acute

## Abstract

Foam rolling (FR) is widely used to acutely enhance range of motion, yet the influence of different application volumes on neuromuscular, structural, and biochemical responses remains unclear. This study investigated the acute effects of low- and high-volume hamstrings FR interventions. Fifty-seven healthy male participants were randomly assigned to a high-volume FR group (IG10; 10 min per posterior thigh), a low-volume FR group (IG2; 2 min per posterior thigh), or a control group (CG; 20 min rest). Pre- and post-intervention assessments included sit-and-reach (SAR) performance, shoulder circumduction (SC) (mobility), maximal voluntary isometric contraction (MVIC), biceps femoris muscle stiffness (MS), and creatine kinase (CK) levels. Linear mixed-effects models revealed significant Time effects for SAR performance and SC, as well as a Group effect for SC (*p* < 0.05), whereas no significant Group × Time interactions were observed for any outcome (all *p* > 0.05). Significant improvements in SC were observed in both intervention groups compared with the CG, whereas no differences were detected between the two intervention durations. In contrast, no differential effects were observed for MS, maximal strength, or CK concentrations between the intervention groups, indicating that extending the FR duration from 2 to 10 min did not provide additional acute benefits.

## 1. Introduction

Foam rolling (FR) has become one of the most widely used self-myofascial massage techniques in sports, fitness, and rehabilitation settings. Due to its simple application, low cost, and minimal equipment requirements, FR is frequently incorporated into warm-up routines [1], recovery protocols [2], and injury-prevention programs [3]. Athletes, coaches, and clinicians commonly use FR with the aim of improving flexibility, enhancing movement quality, reducing muscle soreness, and facilitating recovery following exercise [4]. As a result, the scientific interest in understanding the physiological and performance-related effects of FR has grown substantially over the past decade.

Numerous studies have investigated the acute effects of FR on musculoskeletal and neuromuscular function. Among the most consistent findings is an increase in joint range of motion (ROM) following a single FR session [1,4,5,6]. Meta-analytical evidence indicates that these improvements are comparable to those achieved through traditional stretching interventions [5], while generally avoiding the performance impairments often associated with prolonged static stretching [7,8]. Consequently, FR has emerged as an attractive alternative or adjunct to conventional warm-up strategies.

Beyond improvements in flexibility, FR has been reported to influence several aspects of physical performance. Previous investigations have shown that acute FR interventions generally do not impair maximal strength, power output, or jump performance [9,10], with some studies even reporting small performance enhancements [11,12]. Furthermore, FR has been suggested to positively affect proprioception, neuromuscular control [13,14], and post-exercise recovery [15,16], although findings remain inconsistent across studies [17]. Acute improvements in perceived recovery, reductions in early post-exercise muscle soreness, and short-term increases in stretch tolerance have been reported following single-bout FR interventions [15].

Various classification systems have been proposed to describe the mechanisms underlying massage, myofascial release, and FR. Weerapong et al. [18] distinguished mechanical, physiological, neurological, and psychological effects, whereas other authors propose a simplified separation into mechanical and neurophysiological mechanisms [19,20]. These explanations include thixotropy [19], piezoelectric effects [21], fascial adhesions [22], cellular responses [23], fluid displacement [24], fascial inflammation [25], pain perception [26] and myofascial trigger points [27]. However, there is controversy over foam rolling’s ability to release myofascial adhesions [28].

Despite the growing popularity of FR and the substantial body of literature investigating its acute effects, findings remain inconsistent across outcome measures. While increases in ROM are among the most reproducible findings [5,6], there are also contrary findings [29]. Responses of structural and performance-related parameters are considerably more variable. For example, studies have reported acute reductions in muscle stiffness (MS) following FR [17,30], whereas others have observed negligible or no changes [31,32,33]. Similarly, investigations examining strength, power, proprioception, and neuromuscular function have yielded conflicting results, ranging from small performance enhancements [11] to no measurable effects [10]. These inconsistencies may partly be explained by methodological differences between studies, including the targeted muscle groups, participant characteristics, rolling intensity, pressure application, and outcome measures. Moreover, many studies have focused on a single physiological domain without simultaneously assessing structural and biochemical responses, leaving the relationship between tissue-level alterations and functional outcomes insufficiently understood.

Another less explored aspect concerns the broader neuromuscular and systemic responses to FR. While FR has been shown to acutely affect local ROM [5,34] and strength [35], its potential influence on non-local ROM and strength remains largely unexplored to date [26]. However, unilateral FR presented increases in non-local pain pressure thresholds (decreased pain in contralateral calf) [26].

In addition, it has been suggested that FR interventions may influence systemic markers associated with muscle stress and recovery, such as CK [16]. Although FR is frequently used in practice with the intention of supporting recovery, the extent to which a single FR session can acutely affect biochemical indicators of muscle stress remains unclear. Current findings are inconsistent, and the underlying mechanisms have not yet been sufficiently investigated. As a result, the acute systemic responses to FR represent an emerging research area that warrants further examination.

Among the methodological factors that may contribute to the heterogeneous findings, the volume of FR appears particularly relevant. Existing studies have employed substantially different protocols, ranging from brief interventions lasting less than one minute to multiple sets exceeding five minutes of total rolling duration [36]. Behm et al. (2020) [37] employed regression equations to predict rolling prescriptions for the optimal increase in ROM, suggesting 1–3 sets of 2–4 s repetition duration with a total rolling duration of 30–120 s per set.

Emerging evidence suggests that longer FR durations may induce greater increases in ROM compared with shorter applications, indicating the possibility of a dose–response relationship. However, whether greater FR volumes also produce larger structural, functional, or biochemical adaptations remains largely unknown [7]. Additionally, the cadence of FR, whether performed at a slow or fast rolling speed, may further modulate these effects, as recent evidence suggests that rolling velocity influences neuromuscular and perceptual responses [38].

To date, most studies have examined isolated outcomes following a single FR protocol, making it difficult to determine whether changes in flexibility, tissue properties, and physiological markers are interconnected. Furthermore, the acute effects of different FR volumes on structural, functional, and biochemical responses have never been investigated within the same experimental framework, particularly for the hamstring muscles. As a result, the dose-dependent mechanisms underlying FR-induced adaptations remain largely unresolved.

To the best of our knowledge, the acute effects of hamstrings FR on maximal isometric trunk strength have not yet been investigated. In addition, because the reverse plank position required during the intervention involves continuous activation of the trunk musculature, trunk force production was included as an exploratory outcome. Therefore, the aim of this randomized controlled trial was to investigate the acute effects of a low-volume (2 min) and a high-volume (10 min) FR intervention on Sit-and-Reach (SAR) performance, maximal isometric hamstrings strength, maximal isometric trunk strength, passive hamstrings MS, shoulder circumduction (SC) and CK concentrations in comparison with a passive control condition.

It was hypothesized that the high-volume FR intervention would induce greater acute adaptations than both the low-volume intervention and the passive control condition. Specifically, participants in the high-volume group were expected to demonstrate greater increases in SAR performance, maximal isometric hamstrings strength, and trunk strength, as well as greater reductions in passive hamstrings MS and CK concentrations immediately following the intervention.

## 2. Materials and Methods

### 2.1. Ethical Approval

The study was approved by the Ethics Committee of the University of Graz, Austria (Approval No. 2025/26–7; ID: GZ. 39/7/63 ex 2025/26; approved on 2 December 2025). All procedures were conducted in accordance with the Declaration of Helsinki. Prior to participation, all participants provided written informed consent. The present study was not prospectively registered in a public trial registry.

### 2.2. Participants

An a priori power analysis was performed using G*Power (Version 3.1.9.7; Heinrich Heine University Düsseldorf, Germany) for a repeated-measures analysis of variance (3 groups × 2 time points) investigating the group × time interaction. Assuming a medium effect size (f = 0.25) [6], a significance level of α = 0.05, a statistical power of 0.80, and a correlation among repeated measures of 0.50, the required total sample size was calculated to be 54 participants.

Hence, a total of 57 male participants aged between 20 and 35 were recruited for this study. The inclusion criteria were: (a) male sex and (b) participation in recreational physical activity for at least 60 min per week. Exclusion criteria included neurological disorders, traumatic injuries, muscular disorders, current musculoskeletal injuries affecting the lower limbs, trunk, or shoulder, and any medical condition that could interfere with the testing procedures. The restriction to male participants was chosen to ensure a homogeneous sample and to avoid sex-specific variability in muscle and connective tissue characteristics. In addition, the measurement of the posterior thigh required close physical contact. Therefore, only male participants were included to ensure participant privacy and to address ethical considerations associated with the testing procedures. The exclusion criteria included: (a) having any neurological, traumatic or muscular disorder. The anthropometrics of the participants are presented in Table 1.

### 2.3. Study Design

**Figure 1:**
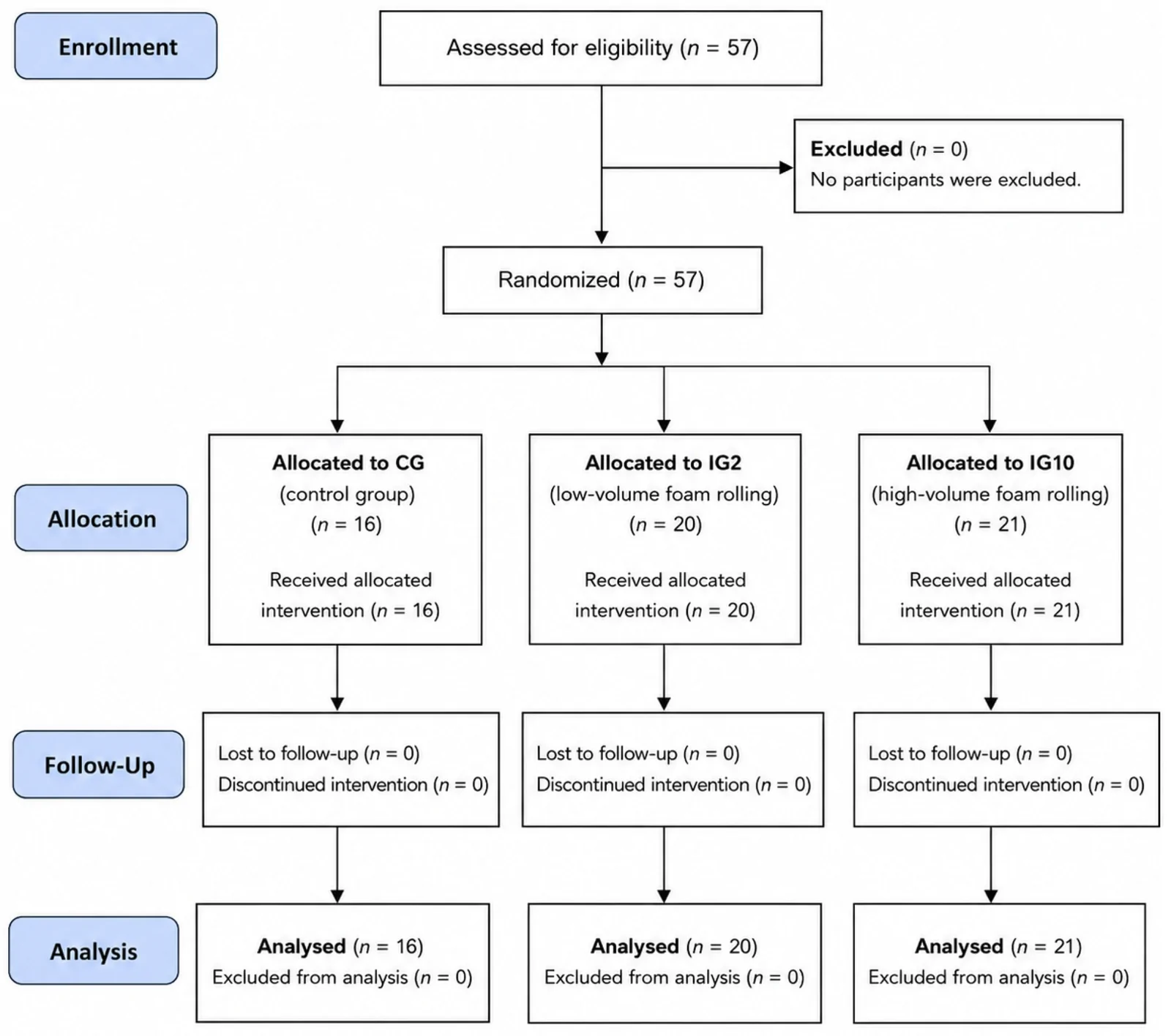
CONSORT study flow diagram. IG2 = low volume foam rolling group; IG10 = high volume foam rolling group; CG = control group; *n* = number of participants.

A randomized controlled study in a parallel design was used to investigate the acute effects of different FR volumes applied to the hamstring muscles. Participants were randomly allocated (i.e., via Randomgroup.org) to either IG10 (*n* = 21; 10 min of rolling per posterior thigh), IG2 (*n* = 20; 2 min of rolling per posterior thigh), or CG (*n* = 16), which rested in a seated position for 20 min to match the total intervention duration of the IG10 group (Figure 1). A randomization sequence for 60 participants was generated using Randomgroup.org with an intended allocation ratio of 1:1:1. Although the required sample size was 54 participants, recruitment was stopped after 57 participants had been enrolled, resulting in final group sizes of 16, 20, and 21 participants according to the predefined randomization sequence. The FR intervention was applied using a standardized technique in a reverse plank position with a proximal-distal rolling cadence of three seconds in each direction, unilateral but on both Hamstrings. The following sections describe the assessments in the order in which they were performed.

### 2.4. Procedures

#### 2.4.1. Pre-Test Protocol

Prior to testing, participants completed standardized anthropometric measurements (height and weight), as well as a warm-up consisting of 5 min of cycling on an ergometer (Ergoselect 4, ergoline GmbH, Bitz, Germany) at a workload relative to body weight (i.e., watts = body weight). Before each testing session, participants received standardized written instructions and were asked to refrain from any sporting or strenuous physical activity on the day before and on the day of testing, maintain their habitual dietary intake, consume their last meal at least 60 min (ideally 120 min) before testing, and attend the testing session only if they were free from acute illness or injury.

#### 2.4.2. Muscle Stiffness

MS of the biceps femoris (BF) was assessed using the MyotonPRO^®^ device (Myoton AS, Tartu, Estonia). This device provides a non-invasive and reliable method for evaluating the biomechanical and viscoelastic properties of skeletal muscle [39]. MS is defined as the mechanical resistance within the muscle tissue and is expressed in Newtons per meter (N/m) [40].

For the measurement, participants were positioned in a prone lying position with their arms relaxed alongside the body and their feet extending beyond the edge of the examination table. The measurement site of the BF was marked at the midpoint (50%) between the ischial tuberosity and the fibular head along the muscle belly. The MyotonPRO^®^ probe was positioned perpendicular to the skin surface, and three consecutive mechanical impulses (0.42 N) were applied to induce tissue oscillation and assess mechanical response.

At each measurement site, three repeated measurements (nine impulses in total) were performed. The mean value of these three trials was used for statistical analysis, consistent with previous research protocols [41].

#### 2.4.3. Sit-and-Reach Test

Hamstrings and lower back flexibility were assessed using the SAR test, a standardized field test for evaluating flexibility of the posterior kinetic chain. A SAR Trunk Flexibility Box (Baseline, Pirmasens, Germany) was used for data collection.

Participants were seated on the floor with hip flexion, fully extended knees, and ankles positioned at 90° dorsiflexion. The feet were placed flat against the box and fixed in position. In the starting position, participants maintained an upright seated posture with both arms extended forward parallel to the floor, and the index fingers touching. For the test execution, participants were instructed to slowly flex forward at the hip and reach as far as possible along the measurement scale using both hands simultaneously. Knee flexion or unilateral reaching movements were not permitted; trials with visible compensatory movements of the trunk or lower limbs were discarded and repeated. To minimize stretch reflex activation, all movements were performed in a slow and controlled manner [42]. Each participant completed three trials with 15 s of rest between attempts. The mean value of the three measurements was used for statistical analysis [42].

#### 2.4.4. MVIC Leg Flexion

MVIC strength of the hamstrings of the dominant leg was assessed using a unilateral leg flexion test on a Compass 540 leg extension/curl machine (Proxomed, Alzenau, Germany).

Joint angles were standardized using a goniometer, with both hip and knee joints set to 110° (180° = anatomical neutral position). The resistance pad was positioned proximal to the calcaneus on the posterior aspect of the Achilles tendon. The non-dominant leg remained relaxed and hanging freely throughout testing to minimize compensatory movements.

Participants were instructed to maintain a stable upper body position with arms crossed over the chest. After a start signal, participants waited for 2 s before gradually increasing force against the isometric resistance. Between seconds 4 and 8, participants exerted maximal voluntary effort.

Each participant performed three maximal isometric contractions with the dominant leg. Each contraction lasted 5 s and was separated by 45 s of rest. Participants were instructed to produce maximal force during each trial. Prior to testing, a familiarization procedure including submaximal and maximal practice contractions with the non-dominant leg was conducted to reduce learning effects and ensure valid force production. For statistical analysis, the highest recorded peak torque value was used [43].

#### 2.4.5. MVIC Trunk Flexion

Maximal voluntary isometric strength of the trunk flexor muscles was assessed using a Compass 540 trunk flexion/extension machine (Proxomed, Alzenau, Germany). Participants were positioned in a seated posture with hip flexion set to 120° (180° = anatomical neutral position). The trunk pad was positioned at approximately the level between the xiphoid process and the nipple line, and the seat height was adjusted to its maximum position. The upper body was stabilized in a physiological double S-shaped spinal alignment. The arms were crossed over the chest with the trunk pad placed between the forearms and the chest. The legs remained relaxed and hanging freely throughout the measurement to avoid additional stabilization or compensatory movements. The resistance lever was set to an angle of 120° (180° = anatomical neutral position).

After a start signal, participants waited for 1 s before gradually increasing force against the isometric resistance. Force was applied in a controlled and progressive manner, reaching maximal effort at second 5 and being maintained until second 9. This gradual increase was intentionally implemented because a sudden maximal contraction causes the dynamometer to overshoot, producing an artificially inflated peak before stabilizing at the true maximal value. To avoid this measurement artefact, participants were instructed to progressively increase force before reaching maximal effort.

Each participant completed three MVIC trials, each lasting 4 s, with 45 s of rest between trials. Participants were instructed to exert maximal effort during each contraction. Prior to testing, participants were familiarized with the protocol. For statistical analysis, the highest recorded peak torque value was used [43].

#### 2.4.6. Bilateral Shoulder Circumduction (SC)

Shoulder mobility was assessed using a bilateral SC test, a standardized method for evaluating global ROM of the glenohumeral joint and functional mobility of the shoulder girdle.

A custom-built 150 cm wooden stick (diameter: 2.5 cm) with 1 cm interval markings was used for measurement. Participants stood in an upright position with feet parallel and held the stick with both hands in front of the body using the widest pain-free grip width. The starting position required full elbow extension, a neutral spinal posture, and equal weight distribution between both lower limbs.

For test execution, participants were instructed to move the stick in a slow, controlled motion overhead and, without elbow flexion or compensatory trunk movement, lower it behind the body. Compensations such as increased lumbar extension, lateral trunk flexion, or widening of the grip width during movement were not permitted; trials with visible compensatory strategies were discarded and repeated.

After each successful attempt, grip width was progressively reduced until the smallest pain-free and technically correct grip width was identified. A smaller grip width reflects greater shoulder mobility. To minimize reflexive muscle activation and jerky movements, all trials were performed at a controlled, moderate velocity [44]. Participants completed multiple trials until the minimal achievable grip width was reached. The smallest successfully performed grip width was used for statistical analysis.

#### 2.4.7. Creatine Kinase

CK concentration was assessed via minimally invasive capillary blood sampling from the earlobe. Blood sampling was performed using UNISTIK 3 EXTRA 21G safety lancets (Owen Mumford GmbH, Großostheim, Germany), providing a standardized and safe skin puncture procedure. The first few blood drops were discarded to minimize potential contamination by interstitial fluid, after which 20 µL of capillary blood was collected for analysis.

CK concentration was analyzed using a SimplexTAS 101 Analyzer (TASCOM Co., Ltd., Anyang-si, Republic of Korea) with manufacturer-specific SimplexTAS 101 CK reagents. The analyzer operates according to a photometric measurement principle, in which CK activity is determined through an enzymatic reaction based on the conversion of creatine phosphate and adenosine diphosphate (ADP) into creatine and adenosine triphosphate (ATP). The generated ATP is subsequently utilized in a coupled reaction, and the resulting change in optical density is detected by the analyzer. Signal intensity is proportional to CK activity in capillary whole blood. Data analysis was performed automatically, and CK concentration was expressed in U·L^−1^.

#### 2.4.8. Foam Rolling Intervention

**Figure 2:**
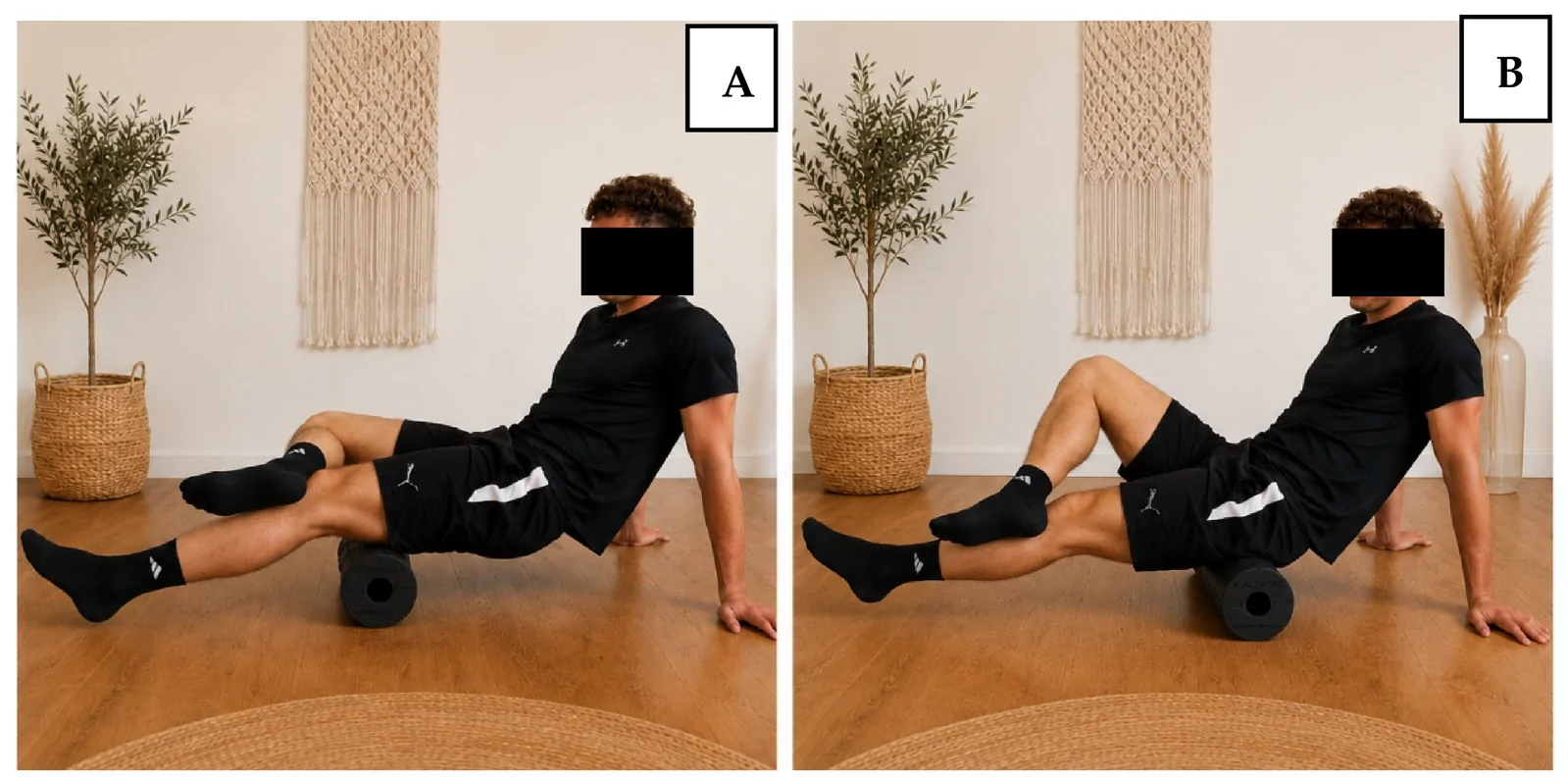
Starting position and turning points during the foam rolling intervention: (**A**) starting position and turning point located near the knee; (**B**) second turning point positioned below the ischial tuberosity (tuber ischiadicum).

The hamstring muscle group was rolled in a reverse plank position with the contralateral leg crossed over, as illustrated in Figure 2. The intervention was performed for two minutes per posterior thigh in IG2 and for ten minutes per posterior thigh in IG10. A proximal-to-distal rolling technique was applied, ensuring continuous pressure along the entire muscle length.

To standardize movement cadence, a metronome (Smart Metronome; Tomohiro Ihara, Japan) was used, set to a 3/0/3/0 rhythm (3 s proximal-to-distal phase/0 s transition/3 s distal-to-proximal phase/0 s transition), corresponding to 20 cycles per minute.

In IG2, the total intervention duration was four minutes, with two minutes of FR performed on the non-dominant posterior thigh followed by two minutes on the dominant posterior thigh. In IG10, the total intervention duration was 20 min, consisting of 10 min per posterior thigh, starting with the non-dominant and followed by the dominant posterior thigh. In both intervention groups, FR pressure (intensity) was relative to the individual’s body mass. The extent of pressure could be manipulated to some extent (body mass could be partially supported with the upper limbs) and participants were asked to perform FR at an individually tolerable intensity, corresponding to approximately 7/10 on a visual analogue scale (VAS), defined as rolling until the point of discomfort but not pain.

### 2.5. Statistical Analysis

All statistical analyses were performed using JASP (version 0.19.8.1; JASP Team, Amsterdam, the Netherlands). Data are presented as means ± standard deviations (SD). Baseline differences between the three groups were assessed using one-way analysis of variance (ANOVA) for normally distributed variables and the Kruskal–Wallis test for CK concentrations, which did not meet the assumption of normality.

Intervention effects were analyzed using linear mixed-effects models fitted by restricted maximum likelihood. Separate models were calculated for each outcome variable. The models included the fixed effects of Time (pre vs. post), Group (CG, IG2, and IG10), and the Group × Time interaction. Participant identification was included as a random intercept to account for the dependency of repeated measurements within participants. Fixed effects were evaluated using Type III tests with Satterthwaite approximations for the degrees of freedom.

The Group × Time interaction was considered the primary test of whether changes over time differed between the three groups. Where a significant interaction was observed, pairwise comparisons with a paired (within-group differences) or unpaired (between-group differences) *t*-test were conducted. Statistical significance was set at *p* < 0.05.

## 3. Results

Baseline differences between the three groups were assessed prior to the intervention. Variables that met the assumption of normality (Shapiro–Wilk test) were analyzed using one-way ANOVA, whereas CK concentrations were analyzed using a Kruskal–Wallis test. No significant baseline differences were observed for MS (*p* = 0.284), SAR performance (*p* = 0.070), MVIC of the hamstrings (*p* = 0.501), MVIC of the trunk flexors (*p* = 0.555), or CK concentrations (*p* = 0.056). However, a significant baseline difference was found for SC (F(2,54) = 4.768, *p* = 0.012). The effects of the intervention were subsequently analyzed using linear mixed-effects models including the fixed effects of Time (pre vs. post-intervention), Group (CG, IG2, and IG10), and the Group × Time interaction. Mean ± SD values for all outcome variables at baseline and post-intervention are presented in Table 2, whereas the results of the linear mixed-effects models are presented in Table 3.

Linear mixed-effects models revealed significant main effects of Time for SAR performance (F(1,54) = 8.374, *p* = 0.005) and SC (F(1,54) = 9.152, *p* = 0.004). A significant main effect of Group was observed only for SC (F(2,54) = 4.778, *p* = 0.012). No significant Group × Time interactions or other Time or Group effects were detected for any outcome variable (all *p* > 0.05).

Post hoc test for SC revealed significantly greater improvements in both intervention groups compared with the control group (CG vs. IG2: *p* < 0.001; CG vs. IG10: *p* < 0.001), whereas no significant difference was observed between IG2 and IG10 (*p* = 0.811).

## 4. Discussion

The present randomized controlled trial investigated whether different volumes of hamstrings FR produce different acute structural, functional, and biochemical adaptations. Contrary to our hypothesis, no significant Group × Time interactions were observed for any outcome variable, indicating that neither the low-volume (IG2) nor the high-volume (IG10) intervention produced treatment-specific effects compared with the CG. Significant main effects of Time were observed for SAR performance and SC, whereas no significant effects were found for passive hamstrings MS, maximal isometric hamstring strength, maximal isometric trunk flexor strength, or CK concentrations. Overall, these findings do not support the existence of a dose–response relationship between the investigated FR volumes. A significant main effect of Time was observed for SAR performance, indicating an overall improvement in flexibility from baseline to post-intervention across all groups. However, the absence of a significant Group × Time interaction suggests that these improvements did not differ significantly between the low-volume FR, high-volume FR, and control groups. Therefore, the present findings do not provide evidence for a treatment-specific effect of either FR protocol on acute ROM. These findings should be interpreted in the context of the existing literature, which consistently reports acute improvements in ROM following FR interventions [1,5,6]. While previous studies and meta-analyses have demonstrated beneficial effects of FR on flexibility, most compared pre- and post-intervention changes within the intervention condition or against passive controls and did not specifically investigate dose–response relationships using a randomized Group × Time framework. Contrary to our hypothesis, the present study did not demonstrate that a longer FR duration resulted in greater ROM improvements. This finding is consistent with a previous study [1], which reported comparable ROM increases following 90 s and 300 s FR interventions, suggesting that extending the duration of FR does not necessarily produce larger acute flexibility gains. Within the investigated volume range, the present findings do not provide evidence that increasing FR duration from 2 to 10 min per posterior thigh results in greater acute adaptations. Considering the 95% CI and near-significant changes (*p* = 0.09) in SAR performance (see Table 2) of the CG (95% CI 0.1 to 1.7) and the IG2 group (95% CI 1.7 to 3.3), possible individual differences might be inferred, although the group effect showed only a trend of a significant change (see Table 3).

No change in SAR performance occurred without significant changes in passive hamstring MS. This finding is consistent with several previous studies reporting no significant alterations in MS following acute FR interventions [32,33,45]. Previous studies suggest that acute improvements in ROM, when observed following FR interventions, are thought to be primarily mediated by increased stretch tolerance rather than substantial alterations in the mechanical properties of the muscle–tendon unit [1,42]. Neurophysiological mechanisms, such as altered pain perception and increased tolerance to stretching discomfort, may therefore enable individuals to reach greater end-range positions during the SAR test. Although small reductions in MS were descriptively observed in both intervention groups, neither the main effects nor the Group × Time interaction reached statistical significance. Therefore, the present findings do not provide evidence that acute improvements in ROM were accompanied by measurable changes in passive hamstrings MS. Furthermore, extending the FR duration from 2 to 10 min on the posterior thigh did not result in greater changes in either ROM or MS, providing no evidence for a dose–response relationship within the investigated intervention volumes.

A significant main effect of Time was observed for SC, indicating an overall improvement in shoulder mobility from baseline to post-intervention across all groups. In addition, a significant main effect of Group was detected. Post hoc analysis revealed a significant favourable effect for SC increases in IG 2 and IG 10 compared to the CG. SC was included as an exploratory outcome to investigate whether the intervention elicited effects beyond the directly treated muscle group. However, these findings should be interpreted with caution, as the intervention itself required continuous weight-bearing through the upper limbs and repeated shoulder movements while maintaining the reverse-plank position [46]. Recent evidence further suggests that such responses should not be attributed exclusively to FR. A previous study [29] reported comparable improvements in passive hip flexion and ankle dorsiflexion ROM following both hamstrings FR and a sham rolling condition, with no significant differences between conditions, suggesting that movement-related warm-up effects may largely explain these acute ROM changes. Similarly, a systematic review and meta-analysis concluded that FR and stretching do not provide superior acute improvements in ROM or MS compared with other warm-up activities [31]. Consequently, the overall improvement in SC observed in the present study is likely explained, at least in part, by movement- and position-related warm-up effects associated with the reverse-plank position rather than by systemic or non-local effects of FR. Throughout the intervention, participants continuously supported part of their body weight with the upper limbs while repeatedly moving the shoulders through extension and flexion, exposing the shoulder complex to dynamic loading and repeated end-range movements similar to those performed during dynamic stretching [47]. Therefore, the observed changes in SC should not be interpreted as evidence of treatment-specific non-local adaptations induced by FR.

Contrary to our hypothesis, neither the low-volume nor the high-volume FR intervention resulted in significant changes in MVIC LF or MVIC TF performance. Furthermore, no significant main effects of Time or Group, and no significant Group × Time interactions, indicating that FR volume had no meaningful influence on maximal isometric strength. These findings are consistent with previous literature showing that acute FR interventions generally do not enhance or impair force production [9,10].

The absence of strength improvements despite greater improvements in SC is noteworthy and supports previous findings suggesting that acute FR may enhance flexibility-related outcomes without affecting neuromuscular performance. While FR may alter sensory perception and increase stretch tolerance, these adaptations do not appear sufficient to influence maximal voluntary force production. Importantly, unlike prolonged static stretching, which has been associated with temporary reductions in strength performance [8], FR appears to preserve strength capacity while improving flexibility-related outcomes.

In contrast to our initial hypothesis, the present study did not reveal a significant Group × Time interaction for CK concentrations, indicating that the immediate changes in CK did not differ between the low-volume FR, high-volume FR, and control groups. Likewise, no significant main effects of Time or Group were observed. These findings suggest that neither a 2 min nor a 10 min FR intervention induced differential acute changes in CK concentrations.

Other CK studies typically employed exercise-induced muscle damage protocols and delayed follow-up assessments of FR on recovery-related outcomes and delayed markers of muscle damage [15,16]. However, CK concentrations typically peak several hours after muscle loading. Since CK was assessed immediately after the intervention and no standardized muscle-damaging exercise protocol preceded the measurements, the present findings should not be interpreted as evidence of enhanced recovery or reduced muscle damage. Rather, they indicate that acute FR did not elicit measurable immediate changes in CK concentrations compared with passive recovery.

Future studies should combine standardized muscle-damaging exercise protocols with repeated follow-up measurements over several hours or days to better characterize the temporal response of CK following different FR volumes.

In summary, the present study found no evidence that acute changes differed between the low-volume FR, high-volume FR, and control groups, as no significant Group × Time interactions were observed for any outcome variable. Significant main effects of Time were detected for SAR performance and SC, indicating overall improvements from baseline to post-intervention across all groups. Furthermore, a significant main effect of Group was observed for SC. Post hoc analyses demonstrated significantly greater improvements in both intervention groups compared with the CG, whereas no significant difference was observed between IG2 and IG10. In contrast, no significant effects were observed for passive hamstrings MS, MVIC hamstrings strength, MVIC trunk flexor strength, or CK concentrations. Furthermore, the present findings do not provide evidence that increasing FR duration from 2 to 10 min per posterior thigh results in greater acute adaptations, thereby providing no support for a dose–response relationship within the investigated intervention volumes.

One possible explanation is that the mechanisms underlying acute FR adaptations reach a plateau after a relatively short duration of mechanical stimulation. Once increases in stretch tolerance and sensory modulation have been elicited, extending the duration of FR may not provide additional physiological benefits. This interpretation is consistent with the absence of a dose–response relationship observed in the present study, as no greater acute adaptations were detected following the longer FR protocol across any of the investigated outcome variables.

The findings of the present study have several practical implications for athletes, coaches, and practitioners. First, the present results do not provide evidence that extending the duration of hamstrings FR from 2 to 10 min per posterior thigh results in greater acute adaptations. Therefore, when FR is incorporated into warm-up routines, longer intervention durations may not offer additional benefits within the investigated volume range. Second, neither FR protocol adversely affected maximal isometric strength, suggesting that acute hamstrings FR can be integrated into warm-up routines without compromising force production. Although overall improvements in SAR performance were observed over time, these changes did not differ significantly between the intervention and control groups. Consequently, the present findings do not support recommending one FR duration over another based on the investigated outcomes.

The applied rolling intensity (pressure) was self-selected (some of the body mass rolling pressure could be supported or counterbalanced by the arms) according to individual discomfort, reflecting common practice in sports. However, this approach may have introduced inter-individual variability in applied pressure due to differences in pain perception and tolerance. In the present study, participants were instructed to perform FR at a target discomfort level of approximately 7/10 on the VAS. Previous reviews have suggested that a moderate pressure corresponding to approximately 5/10 on the VAS may provide an effective balance between efficacy and tolerability [4,10]. Therefore, future studies should investigate whether different rolling intensities influence the physiological and functional responses to FR.

Several limitations of the present study should be acknowledged. First, the sample consisted exclusively of healthy young males, which limits the generalizability of the findings to other populations such as females, older adults, clinical populations, or elite athletes. Another limitation relates to participant characterization. Although participants were required to engage in at least 60 min of physical activity per week, detailed information regarding training history, training volume, sport discipline, and competitive level was not collected. Consequently, participants could not be classified according to established participant classification frameworks, such as that proposed by McKay et al. [48], which may limit comparisons with previous studies. Third, previous experience with FR was not assessed. Therefore, differences in familiarity with the technique may have influenced movement execution, pressure application, and participants’ responses to the intervention. Rolling intensity was self-selected according to perceived discomfort but was not quantified using a VAS. Consequently, inter-individual differences in pain perception may have influenced the applied pressure and contributed to the variability of the responses. Further, only the acute effects of a single FR session were investigated. Consequently, the present results cannot be transferred to long-term adaptations resulting from repeated FR interventions, and future research is needed to examine potential chronic effects of different FR volumes. Furthermore, MS was assessed only at a single location of the M. biceps femoris. As FR may affect different regions of the muscle–tendon unit differently, localized measurements may not fully reflect changes throughout the entire hamstrings complex. Another limitation concerns the assessment of SC. Because the intervention was performed in a reverse plank position requiring continuous weight-bearing through the upper limbs and repeated shoulder movements, the observed improvements are more likely explained by movement-related warm-up effects than by non-local effects of FR. Although previous studies have reported contralateral improvements in ROM following unilateral FR, suggesting that non-local neurophysiological mechanisms may exist [49], the present experimental design does not allow such mechanisms to be distinguished from local movement-related effects. Furthermore, SC was the only upper-body outcome assessed, limiting conclusions regarding potential non-local or systemic adaptations. Future studies should include additional remote outcome measures and experimental designs that better isolate non-local effects of FR. Another limitation concerns the assessment of CK concentrations. Since CK typically reaches its peak several hours after muscle loading, measurements obtained immediately after the intervention may not have captured the full biochemical response. Furthermore, participants in the present study did not perform a standardized muscle-damaging exercise protocol prior to the intervention. Therefore, the absence of substantial CK responses should be interpreted with caution and should not be considered evidence regarding the recovery-enhancing effects of FR or its ability to attenuate exercise-induced muscle damage. Likewise, the immediate post-intervention changes in CK should not be interpreted as definitive indicators of enhanced recovery or reduced muscle damage but rather as exploratory biochemical responses. Moreover, the assessment of CK alone may not provide a comprehensive picture of exercise-induced muscle damage and recovery processes. Future research should therefore consider incorporating additional biomarkers, such as lactate dehydrogenase (LDH), myoglobin, inflammatory markers, or cytokines, to obtain a more holistic understanding of the physiological responses to FR.

Finally, although the present study compared two substantially different FR volumes, the rolling cadence was standardized and pressure was applied according to individual tolerance. Since both rolling speed and applied pressure may influence the physiological responses to FR, future studies should investigate the interaction between volume, cadence, and pressure to further optimize FR prescription.

## 5. Conclusions

The present randomized controlled trial investigated the acute effects of low-volume (2 min per leg) and high-volume (10 min per leg) hamstring foam rolling on SAR performance, shoulder mobility, maximal isometric strength, passive muscle stiffness, and CK concentrations. Linear mixed-effects models revealed significant main effects of Time for SAR performance and shoulder mobility. In addition, a significant main effect of Group was observed for shoulder mobility. However, no significant Group × Time interactions were found for any outcome variable. Furthermore, no significant effects were found for passive hamstring MS, maximal isometric hamstring strength, maximal isometric trunk flexor strength, or CK concentrations.

The present findings do not provide evidence that extending hamstring FR from 2 to 10 min per posterior thigh results in greater acute adaptations, providing no support for a dose–response relationship within the investigated volume range. Although a significant main effect of Group was observed for shoulder mobility, both FR protocols resulted in comparable improvements, with no differences between the 2 and 10 min interventions. The absence of changes in passive MS despite overall improvements in flexibility is consistent with the hypothesis that acute increases in ROM are primarily related to altered stretch tolerance rather than changes in the mechanical properties of the muscle–tendon unit. This is consistent with recent evidence in football players, where a plyometric intervention produced no group-level changes in Myoton-derived muscle tension, stiffness, or elasticity despite significant performance gains, with individual variability in muscle elasticity moderating the response [50].

From a practical perspective, increasing the duration of hamstring FR beyond 2 min per posterior thigh did not result in greater acute responses within the investigated outcomes. Furthermore, neither FR protocol adversely affected maximal isometric strength, suggesting that acute hamstring FR can be incorporated into warm-up routines without compromising force production.

## Figures and Tables

**Table 1:** Descriptive statistics of the participants.

Variable	CG (*n* = 16)	IG2 (*n* = 20)	IG10 (*n* = 21)
Age (years)	26.4 (3.4)	24.6 (2.9)	26.1 (3.7)
Height (cm)	178.7 (6.5)	181.1 (5.9)	177.4 (6.2)
Weight (kg)	76.2 (7.2)	80.6 (7.0)	77.2 (6.9)
BMI (kg/m^2^)	23.8 (1.4)	24.5 (1.4)	24.5 (1.9)

Note: Values are presented as M (SD). CG, Control group; IG2, Intervention group 2; IG10, Intervention group 10. BMI = body mass index.

**Table 2:** Descriptive statistics for all outcome variables before and after the intervention.

Variable	Group	Pre-Test		Post-Test		Delta Pre-Post	
		Mean (SD)	[95% CI]	Mean (SD)	[95% CI]	Mean (SD)	[95% CI]
Muscle stiffness (N/m)	CG	335.4 (44.6)	311.7–359.2	337.4 (45.0)	313.4–361.4	2 (5.8)	−1.1–5.1
	IG2	331.6 (34.2)	315.5–347.5	325.9 (36.7)	308.7–343.0	−4.1 (17.8)	−12.4–4.3
	IG10	315.8 (42.2)	296.6–335.0	317.5 (43.6)	297.6–337.3	−7.5 (16.6)	−15.0–0.1
Sit-and-Reach (cm)	CG	21.1 (7.6)	17.1–25.1	21.5 (7.5)	17.5–25.5	0.9 (1.5)	0.1–1.7
	IG2	18.9 (7.8)	15.2–22.5	20.4 (7.6)	16.8–23.9	2.5 (1.7)	1.7–3.3
	IG10	24.5 (14.7)	21.0–28.0	25.2 (8.1)	21.5–28.9	2.0 (1.4)	1.3–2.6
MVIC Leg Flexion (Nm)	CG	108.8 (17.9)	99.2–118.3	108.4 (17.4)	99.2–117.7	−3.4 (6.4)	−6.9–0.0
	IG2	116.6 (13.3)	135.4–164.2	115.0 (16.1)	107.4–122.5	−1.6 (7.8)	−5.3–2.1
	IG10	112.5 (25.4)	101.0–124.0	110.5 (28.1)	97.8–123.3	−0.8 (8.9)	−4.8–3.2
MVIC Trunk Flexion (Nm)	CG	148.9 (32.1)	131.8–166.0	149.4 (36.3)	129.9–168.6	3.1 (9.9)	−2.2–8.4
	IG2	149.8 (30.8)	101.8–114.8	149.8 (29.7)	135.9 −163.9	−3.8 (14.3)	−10.5–2.9
	IG10	158.7 (30.3)	144.9–172.5	152.4 (26.6)	140.3–164.5	0.3 (20.0)	−8.8–9.4
Shoulder circumduction (cm)	CG	98.8 (16.2)	90.1–107.4	98.6 (15.7)	90.3–107.0	−1.0 (1.5)	−1.8–−0.2
	IG2	108.3 (13.9)	101.8–114.8	106.4 (14.8)	99.4–113.3	−3.9 (2.4)	−5.0–−2.7
	IG10	95.2 (12.0)	89.7–100.7	92.8 (12.3)	87.2–98.4	−3.8 (2.9)	−5.1–−2.5
Creatine kinase (U·L^−1^)	CG	296.5 (193.6)	193.3–399.7	292.0 (183.0)	194.5–389.5	−4.0 (21.4)	−15.4–7.4
	IG2	393.0 (291.7)	256.5–529.5	387.6 (298.6)	247.8–527.4	−16.3 (16.4)	−23.9–−8.6
	IG10	240.6 (172.3)	162.2–319.1	239.7 (175.3)	160.0–319.5	7.2 (25.6)	−4.4–18.8

Note: Data are presented as mean ± standard deviation (SD) and 95% confidence intervals (95% CI) at baseline (pre-test), post-intervention (post-test), and as the mean change (Δ = post − pre). MVIC = maximal voluntary isometric contraction; N/m = Newton per meter; Nm = Newton meter; cm = centimeter; U·L^−1^ = units per liter; CG = control group; IG2 = 2 min foam rolling intervention group; IG10 = 10 min foam rolling intervention group.

**Table 3:** Results of the linear mixed-effects model for all outcome variables.

Variable	Time (1, 54)	*p*	Group (2, 54)	*p*	Group × Time (2, 54)	*p*
Sit-and-Reach (cm)	8.37	0.005	2.50	0.091	1.18	0.316
Shoulder circumduction (cm)	9.15	0.004	4.78	0.012	1.85	0.167
Muscle stiffness (N/m)	0.12	0.733	1.15	0.326	1.52	0.229
MVIC Leg Flexion (Nm)	1.45	0.234	0.57	0.568	0.20	0.817
MVIC Trunk Flexion (Nm)	0.93	0.340	0.27	0.761	1.14	0.328
Creatine kinase (U·L^−1^)	0.87	0.356	2.28	0.112	0.14	0.873

Note: N/m, Newton/meter; cm, Centimeter; U·L^−1^, Units per Liter; CG, Control group; IG2, Intervention group 2; IG10, Intervention group 10; Results are presented as F-values and corresponding *p*-values from the linear mixed-effects model for the fixed effects of Time, Group, and the Group × Time interaction. MVIC = maximum voluntary isometric contraction.

## Data Availability

The data is available upon request.

## Abbreviations

CG	Control Group
CK	Creatine Kinase
FR	Foam Rolling
IG2	Intervention Group 2
IG10	Intervention Group 10
LF	Leg Flexion
MS	Muscle Stiffness
MVIC	Maximum Voluntary Isometric Contraction
SAR	Sit-and-Reach
SC	Shoulder Circumduction
SD	Standard Deviation
SFMR	Self-Myofascial Release
TF	Trunk Flexion
VAS	Visual Analogue Scale
